# Jejunal Perforation Associated With a Horseshoe‐Shaped Abdominal Contusion After a Horse Kick: A Case Report

**DOI:** 10.1002/ccr3.73687

**Published:** 2026-10-02

**Authors:** Jun Yoshida, Takehiko Hanaki, Yu Sakano, Yoshiyuki Fujiwara

**Affiliations:** ^1^ Division of Gastrointestinal and Pediatric Surgery, Department of Surgery Tottori University Faculty of Medicine Yonago Japan; ^2^ Division of Medical Education, Department of Medical Education Tottori University Faculty of Medicine Yonago Japan; ^3^ Department of Surgery Tottori Red Cross Hospital Tottori Japan

**Keywords:** blunt abdominal trauma, equestrian trauma, horse kick, isolated small bowel perforation, jejunal injury

## Abstract

A horseshoe‐shaped abdominal contusion after a horse kick may indicate the site of underlying bowel injury. When accompanied by persistent abdominal pain or peritoneal signs, hollow viscus injury should be considered. Contrast‐enhanced CT can identify free air and focal bowel abnormalities at the impact site, facilitating timely surgical management.

## 1 Introduction

A horse kick is generally regarded as a high‐energy mechanism of injury; however, its clinical presentation does not always reflect the severity of the underlying damage [1]. The impact force of a horse kick has been reported to reach up to 7000 N (approximately equivalent to the weight of a 700‐kg mass) and can cause severe complications, including visceral injury [2]. In clinical practice, however, some patients present with deceptively mild initial findings, which may complicate timely diagnosis and appropriate management.

Although routine interaction with horses has become less common in modern societies [3], the risk of horse‐related trauma persists in specific settings such as riding clubs and farms [1].

In blunt abdominal trauma, solid organ injuries such as those involving the liver and spleen are more common than gastrointestinal injuries [4]. Hollow viscus injury is relatively uncommon but carries significant morbidity when present. Among hollow viscus injuries caused by blunt abdominal trauma, the small bowel is the most frequently affected segment, and perforation represents a well‐recognized manifestation in this setting [5, 6]. Nevertheless, diagnosis can be challenging when the injury is isolated and initial clinical findings are mild or nonspecific. Proposed mechanisms of small bowel injury include crushing of the bowel against fixed structures such as the spine, as well as a sudden increase in intraluminal pressure resulting from closed‐loop formation [7].

Abdominal injuries associated with horse kicks are often described in the context of severe multisystem trauma, frequently requiring damage control surgical management [8, 9]. In contrast, isolated small bowel perforation without associated organ injury may present with subtle or nonspecific early clinical signs, making timely recognition difficult without cross‐sectional imaging [10, 11]. A prior case report described jejunal perforation after a horse kick in an adolescent patient, and other reports have described various patterns of small‐bowel injury associated with horse kicks [8, 9, 12].

Given the potential for deceptively mild initial presentation despite a high‐energy mechanism of injury, isolated small bowel perforation following a horse kick may be easily overlooked. We report a case of isolated jejunal perforation without associated solid organ injury and discuss its clinical presentation, diagnostic considerations, and surgical management in the context of the existing literature.

## 2 Case History/Examination

A 69‐year‐old woman was kicked in the left lower quadrant of the abdomen by a horse while opening the door of a horse stable. She experienced persistent abdominal pain following the injury and initially took oral analgesics at home. As her symptoms persisted despite oral analgesics, she was brought to our emergency department by ambulance approximately 3 h after the injury.

On arrival, the patient was alert, with a blood pressure of 148/79 mmHg and a heart rate of 62 beats per minute. Physical examination revealed generalized abdominal tenderness with rebound tenderness and muscular guarding, consistent with diffuse peritoneal irritation. A horseshoe‐shaped contusion was recognized in the left lower quadrant during the initial emergency department examination and was photographed at presentation (Figure 1). No other external signs of trauma, such as lacerations, additional contusions, or fractures, were identified on physical examination. Her medical history was unremarkable.

**FIGURE 1:**
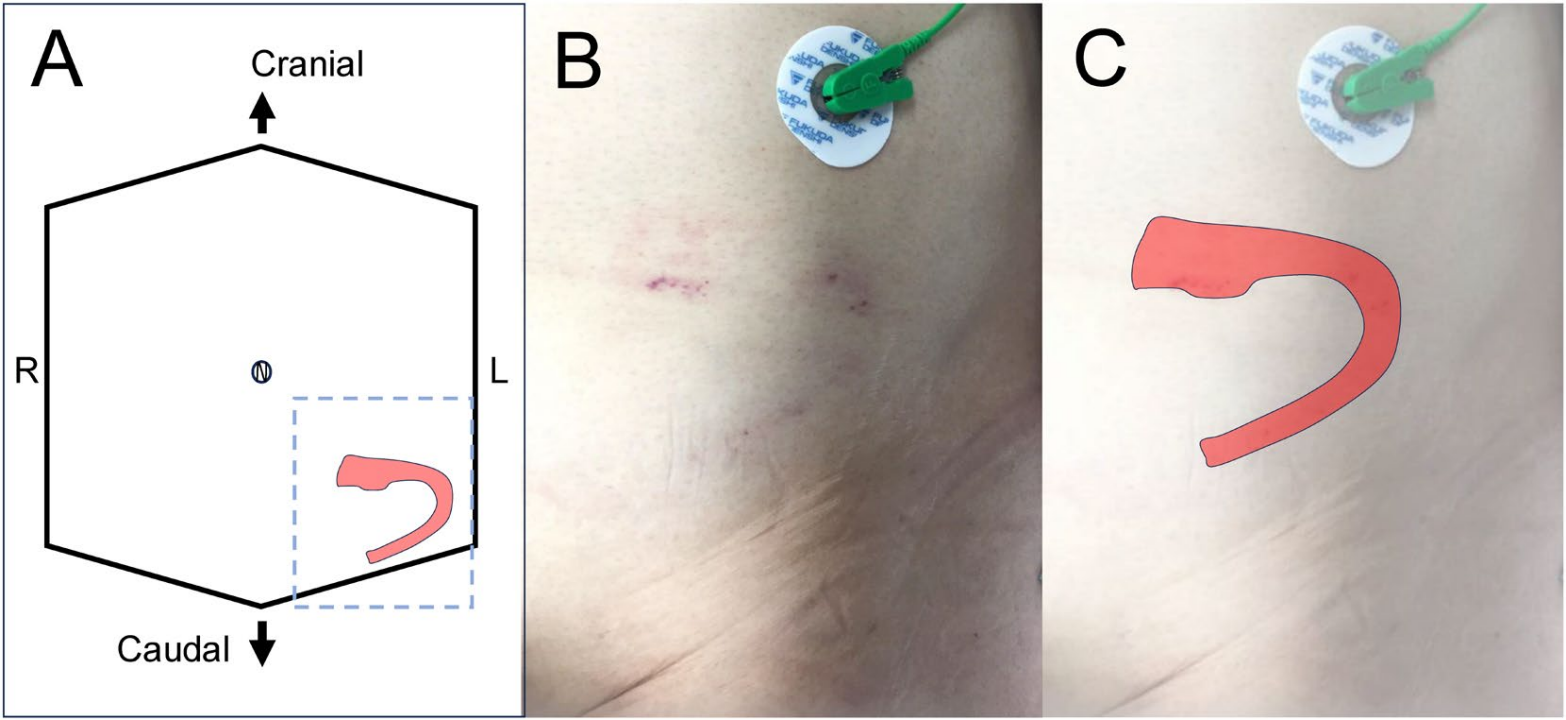
Location and appearance of the abdominal wall contusion. (A) Schematic illustration showing the location of the horseshoe‐shaped contusion in the left lower quadrant of the abdomen. The dashed box indicates the region corresponding to the clinical photograph. (B) Clinical photograph at presentation demonstrating a faint horseshoe‐shaped subcutaneous hemorrhage in the left lower quadrant. (C) Annotated photograph outlining the horseshoe‐shaped contusion.

## 3 Differential Diagnosis, Investigations, and Treatment

Laboratory investigations revealed leukocytosis, with a white blood cell count of 13,400/μL. Hemoglobin level, platelet count, liver enzyme levels, renal function, and C‐reactive protein level were within normal ranges. Focused assessment with sonography for trauma (FAST) was negative. Arterial blood gas analysis showed a lactate level of 2.4 mmol/L and a base excess of −0.9 mmol/L. The Injury Severity Score (ISS) was 9 [13]. Contrast‐enhanced computed tomography of the abdomen demonstrated intraperitoneal free air and focal bowel wall thickening with increased contrast enhancement of the small intestine located directly beneath the abdominal wall contusion (Figure 2A,B). No associated injuries to solid organs or other intra‐abdominal structures were identified.

**FIGURE 2:**
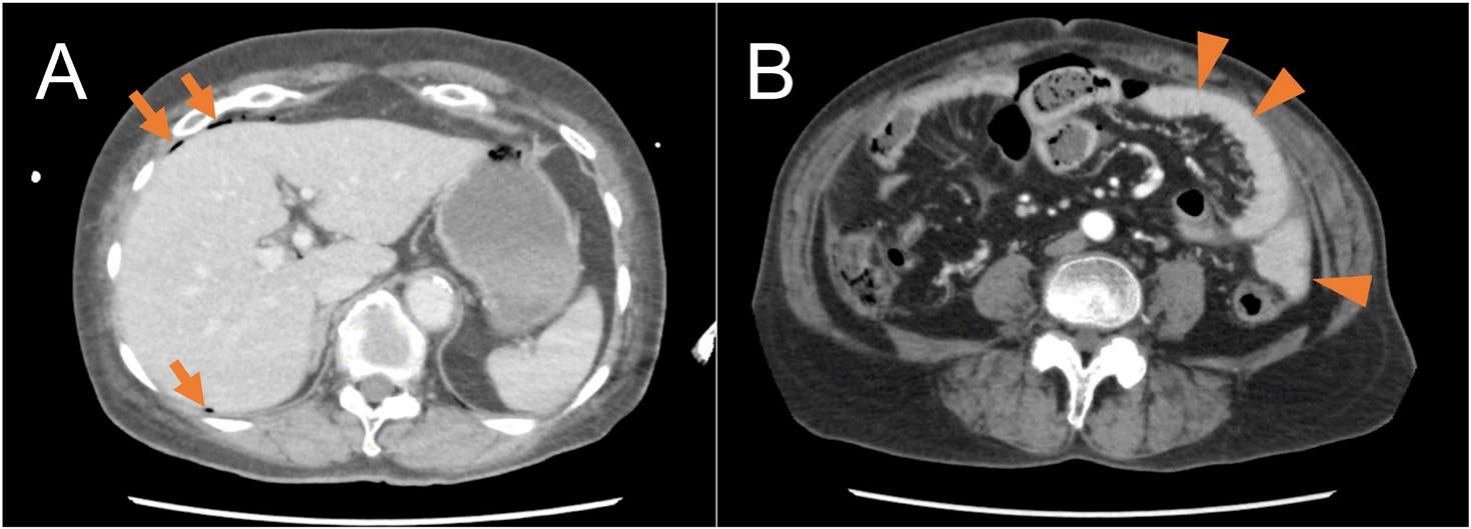
Contrast‐enhanced abdominal computed tomography images. (A) Axial image demonstrating intraperitoneal free air (arrows). (B) Axial image at a lower level showing focal bowel wall thickening and increased enhancement in the jejunum (arrowheads), suggestive of bowel injury, along with swelling of the left abdominal oblique muscle.

Based on the presence of diffuse peritoneal irritation and CT findings of intraperitoneal free air with focal bowel wall thickening directly beneath the abdominal wall contusion in the left lower quadrant, traumatic small bowel perforation near the impact site was considered the leading preoperative diagnosis. Emergency laparotomy was therefore selected and performed approximately 6 h after the injury. A midline incision was made to access the abdominal cavity. Systematic exploration of the entire gastrointestinal tract was performed. Intraoperatively, a fingertip‐sized perforation was identified on the antimesenteric border of the jejunum, approximately 70 cm distal to the ligament of Treitz (Figure 3A). The injury was classified as Grade III according to the American Association for the Surgery of Trauma (AAST) bowel injury scale [14]. No additional injuries to the gastrointestinal tract or other intra‐abdominal organs were identified. The surrounding bowel wall appeared viable, without extensive contusion, ischemia, or mesenteric vascular compromise. The abdominal cavity was irrigated with 10 L of normal saline. After debridement of the edges of the injured bowel wall, the perforation was primarily repaired with simple interrupted 4–0 polydioxanone sutures (Figure 3B). The fascia was closed with #1 polyglactin sutures. The subcutaneous wound was then irrigated with 2 L of normal saline, and the skin was closed with buried subcuticular 4–0 polydioxanone sutures.

**FIGURE 3:**
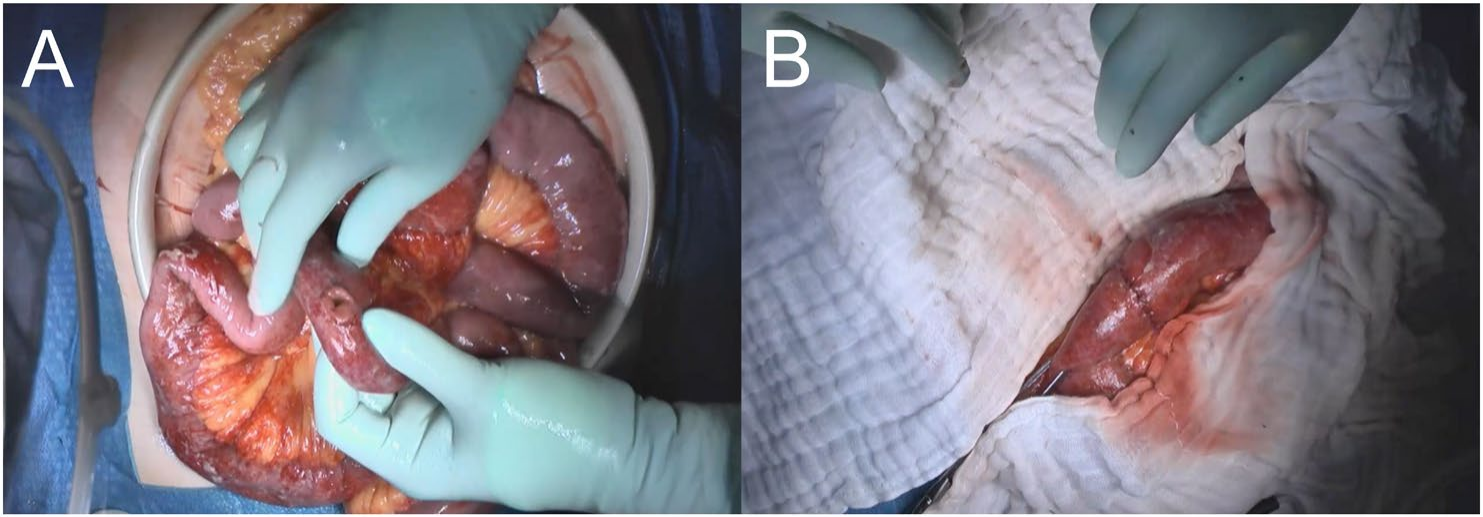
Intraoperative findings. (A) The jejunal perforation was clearly identified on the antimesenteric border. Subserosal hemorrhage was observed in the surrounding small bowel. (B) After trimming the edges of the injured bowel wall, the perforation was repaired by simple closure.

## 4 Outcome and Follow‐Up

The postoperative course was complicated by transient postoperative ileus with delayed return of bowel function. Piperacillin/tazobactam was administered for 7 days. Oral intake was resumed on postoperative day 6. Following gradual dietary advancement and confirmation of stable bowel function, the patient was discharged on postoperative day 11. At a 1‐month follow‐up visit, she reported no abdominal symptoms, including pain or signs of bowel obstruction.

## 5 Discussion

Horse kicks deliver a concentrated high‐energy force over a small contact area. Biomechanical studies have shown that the impact force can reach several thousand N, sufficient to cause bowel perforation through direct compression against fixed retroperitoneal structures or by a sudden increase in intraluminal pressure [2, 7]. In the present case, the anatomical correspondence between the horseshoe‐shaped abdominal wall contusion and the jejunal perforation was consistent with a localized compression mechanism.

In blunt abdominal trauma, solid organ injuries such as those to the liver and spleen are far more common than hollow viscus injuries [15]. Consequently, in patients without obvious hemorrhagic findings, clinical attention may be directed toward parenchymal organs. However, hollow viscus injuries carry substantial morbidity when diagnosis is delayed [4]. In this patient, contrast‐enhanced computed tomography promptly identified intraperitoneal free air without evidence of solid organ injury, directing attention to possible bowel perforation. Emergency laparotomy revealed an isolated fingertip‐sized jejunal perforation with minimal contamination, allowing successful primary closure after trimming the wound edges and contributing to the favorable postoperative course.

The operative approach also warrants consideration. In this case, emergency laparotomy was selected because of diffuse peritoneal irritation and concern for hollow viscus injury. However, in hemodynamically stable patients in whom the suspected injury can be reasonably localized, diagnostic and therapeutic laparoscopy may also be a reasonable option, provided that thorough exploration can be achieved and the threshold for conversion to laparotomy remains low [5, 16, 17].

Isolated small bowel perforation following blunt trauma can present with subtle or nonspecific early findings [11]. Delayed recognition of such injuries has been associated with increased postoperative complications and mortality [18, 19]. These facts highlight the need for a high index of suspicion in patients sustaining high‐energy blunt abdominal trauma. In cases of horse kicks, early contrast‐enhanced computed tomography should be considered when abdominal pain or peritoneal signs persist, even if external findings appear localized.

Previous reports have described various patterns of small‐bowel injury following horse kicks, including jejunal perforation and more complex injuries involving the mesentery and abdominal wall [8, 9, 12]. The present case is notable in that the jejunal perforation was isolated, anatomically corresponded to a horseshoe‐shaped abdominal contusion, and was identified early by contrast‐enhanced CT, allowing primary repair before extensive contamination developed.

This case reinforces an important principle in trauma care: localized external findings do not reliably exclude clinically significant intra‐abdominal injury when abdominal pain or peritoneal signs persist. After a high‐energy localized impact, early cross‐sectional imaging and timely surgical intervention are essential to prevent missed injuries and optimize patient outcomes.

In summary, horse kicks may cause isolated but surgically significant hollow viscus injury, even when external findings are localized and solid organ injury is absent. Persistent abdominal pain or peritoneal signs after high‐energy localized impact should prompt early imaging and timely surgical management when bowel injury is suspected.

## Author Contributions

**Jun Yoshida:** conceptualization, data curation, investigation, writing – original draft. **Takehiko Hanaki:** conceptualization, investigation, writing – original draft, writing – review and editing, resources. **Yu Sakano:** writing – review and editing, investigation, resources. **Yoshiyuki Fujiwara:** writing – review and editing, supervision.

## Funding

The authors have nothing to report.

## Ethics Statement

Ethics approval was waived for this case report in accordance with institutional policy.

## Consent

Written informed consent was obtained from the patient for publication of this case report and accompanying images. The consent obtained included permission for publication of clinical information and photographic material on an open‐access basis.

## Conflicts of Interest

The authors declare no conflicts of interest.

## Data Availability

Data sharing does not apply to this article because no datasets were generated or analyzed. Relevant clinical information and images are included in this article.
